# Genetic characterization and pathogenicity of a Eurasian avian-like H1N1 swine influenza reassortant virus

**DOI:** 10.1186/s12985-022-01936-6

**Published:** 2022-12-02

**Authors:** Hechao Zhu, Xiangmin Li, Huanchun Chen, Ping Qian

**Affiliations:** 1 State Key Laboratory of Agricultural Microbiology, Huazhong Agricultural University, Wuhan, 430070 Hubei China; 2 College of Veterinary Medicine, Huazhong Agricultural University, Wuhan, 430070 Hubei China; 3 Key Laboratory of Preventive Veterinary Medicine in Hubei Province, The Cooperative Innovation Center for Sustainable Pig Production, Wuhan, 430070 Hubei China

**Keywords:** Next-generation sequencing (NGS), Swine influenza, Eurasian avian-like H1N1 virus, Phylogenetic analysis, Pathogenicity

## Abstract

**Background:**

Swine influenza viruses (SIV), considered the “mixing vessels” of influenza viruses, posed a significant threat to global health systems and are dangerous pathogens. Eurasian avian-like H1N1(EA-H1N1) viruses have become predominant in swine populations in China since 2016.

**Methods:**

Lung tissue samples were obtained from pregnant sows with miscarriage and respiratory disease in Heilongjiang province, and pathogens were detected by Next-generation sequencing (NGS) and PCR. The nucleic acid of isolates was extracted to detect SIV by RT-PCR. Then, SIV-positive samples were inoculated into embryonated chicken eggs. After successive generations, the isolates were identified by RT-PCR, IFA, WB and TEM. The genetic evolution and pathogenicity to mice of A/swine/Heilongjiang/GN/2020 were analyzed.

**Results:**

The major pathogens were influenza virus (31%), Simbu orthobunyavirus (15%) and Jingmen tick virus (8%) by NGS, while the pathogen that can cause miscarriage and respiratory disease was influenza virus. The SIV(A/swine/Heilongjiang/GN/2020) with hemagglutination activity was isolated from lung samples and was successfully identified by RT-PCR, IFA, WB and TEM. Homology and phylogenetic analysis showed that A/swine/Heilongjiang/GN/2020 is most closely related to A/swine/Henan/SN/10/2018 and belonged to EA-H1N1. Pathogenicity in mice showed that the EA-H1N1 could cause lethal or exhibit extrapulmonary virus spread and cause severe damage to respiratory tracts effectively proliferating in lung and trachea.

**Conclusion:**

A/swine/Heilongjiang/GN/2020 (EA-H1N1) virus was isolated from pregnant sows with miscarriage and respiratory disease in Heilongjiang province, China. Clinical signs associated with influenza infection were observed during 14 days with A/swine/Heilongjiang/GN/2020 infected mice. These data suggest that A/swine/Heilongjiang/GN/2020 (EA-H1N1) had high pathogenicity and could be systemic spread in mice.

**Supplementary Information:**

The online version contains supplementary material available at 10.1186/s12985-022-01936-6.

## Introduction

Swine influenza A viruses (SIV) belong to the genus influenza A viruses (IAV) of the family Orthomyxoviridae and are dangerous pathogens for pigs compared to influenza B, C, and D viruses [1, 2]. The hemagglutinin (HA) and neuraminidase (NA) proteins of the IAV are applied to further classify them into subtypes [3]. Although there are currently 18 HA subtypes and 11 NA subtypes, most of which circulate in wild birds, antigenic variation of influenza arises rapidly from drift and shift resulting in the creation of novel IAV [4].

Pigs are regarded as “mixing vessels” for the production of IAV with pandemic potential because they have both SA2,3Gal and SA2,6Gal receptor residues dispersed throughout the respiratory tracts, whereas avian, swine and human IAV can undergo genetic reassortment [5, 6]. Once the novel IAV is established in pigs, it will be dangerous to the animals and pose a risk of spreading to people and causing outbreaks and pandemics with introduction or reintroduction into humans [7]. The emergence of the pandemic 2009 H1N1 (pdm09) viruses, which contains genetic fragments from avian, swine, and human, further emphasizes the risk by highlighting the swine in the production of influenza reassortant variants that could infect humans [8].

With avian/human, human/swine, and human/avian/swine triple-reassortant (TP) lineage, Eurasian avian-like (EA) lineage SIV, and classical swine (CS) lineage SIV cocirculating in pigs, China possesses the most complicated SIV ecosystem [9]. Based on lineage classification, six genotypes of G1–G6 EA reassortant were found in EA H1N1 viruses from 2011 to 2018 [2]. The predominant emergent EA reassortant viruses (EA-H1N1) in pigs have pdm09 and TPs-derived internal genes and have shown a sharp increase since 2016 [2]. EA-H1N1 is the predominant genotype in circulation in pigs detected across at least 10 provinces and possesses all of the essential hallmarks of a candidate pandemic virus [10–12]. Therefore, understanding the evolution and pathogenicity of EA-H1N1 in China may help to guide management and pandemic preparedness strategies [2].

In this study, we phylogenetically characterized SIV isolates obtained from pregnant sows with miscarriage and respiratory disease on farms in Heilongjiang, China. Additionally, to find out the virulence of the virus in mammals, the mouse mode was used to study the pathogenicity of the isolate.

## Materials and methods

### Nucleic acid extraction and pathogen detection

3 tissue samples including heart, lung, placenta, submaxillary lymph nodes and tonsil from miscarriage and respiratory disease in pregnant sows were collected at a period (September 2020 and October 2020) on the same pig farm in Heilongjiang province. Tissue samples were homogenized and diluted with sterile PBS, collected supernatant followed by centrifugation at 8000 g for 10 min at 4 °C to remove residual tissue debris. Next-generation sequencing (NGS) was used to detect pathogens that miscarriage and respiratory disease in pregnant sows or other common clinical pathogens. RNA was extracted using TRIzol reagent accordance with the manufacturer’s protocol. For RNA NGS, cDNA library was constructed using TR503-01 reagents according to the manufacturer’s instructions, and an oligonucleotide of known sequence was added to the sample for quality control. Sequencing libraries were generated using the Nextera XT DNA Library Prep Kit (Illumina) with dual index pairs. Libraries underwent amplification with the following conditions as previously reported [13]: (i) 72 °C for five minutes and 95 °C for 30 s, (ii) up to 25 cycles of 95 °C for 15 s, 55 °C 30 s, and 72 °C for 60 s, (iii) 72 °C for 5 min. Then, the libraries were quantified using the KAPA universal complete kit (Roche, Swiss Confederation), pooled to equal concentration, and sequenced on an Illumina platform using paired-end 100 or 150 bp reads.

### Isolation and identification of SIV

SIV was isolated and expanded in embryonated chicken eggs. SIV-positive samples were filtered using 0.22 μm filters and then inoculated into 10-day-old embryonated chicken eggs at 37 °C for 72 h as previously described [2, 14]. At 4 days post-infection (dpi), the virus was harvested and then inoculated into fresh embryonated chicken eggs. After successive generations, viral RNA was extracted. Reverse transcription was carried out using the Uni12 (AGCAAAAGCAGG) primer to detect SIV by RT-PCR [15]. The virus was identified by RT-PCR using specific primers M1 gene of SIV (Additional file 1: Table S1). The subtype of the virus was identified by RT-PCR using subtype-specific primers [16, 17]. After 3 generations, the virus was harvested and then infected MDCK (Madin-Darby canine kidney) cells containing 2 µg/mL TPCK-trypsin. MDCK cells were cultured in high-glucose DMEM (Dulbecco’s modified Eagle medium, HyClone, Marlborough, MA, USA) supplemented with 10%(v/v) FBS (heat-inactivated fetal bovine serum, Inner Mongolia opcel Biotechnology Co., Ltd., Inner Mongolia, China) for 50% tissue culture infective dose (TCID_50_) and Indirect immunofluorescence assay (IFA). 1% chicken erythrocytes were prepared from EDTA anticoagulant and Alsever’s Solution to determine hemagglutination activity.

IFA was performed to detect viral activity of isolates. Briefly, Confluent with MDCK cells in 24-well plates were washed three times with PBS and infected with the isolates at 0.1 MOI in serum-free DMEM containing 2 µg/mL TPCK-trypsin for 16 h. The infected MDCK cells were washed 3 times with phosphate buffer containing Tween 20 (PBST) and then fixed with 4% paraformaldehyde solution for 30 min at 4 °C. The blocked cells were incubated with NP monoclonal antibody (Abcam, UK) for 2 h at 37 °C. Subsequently, the cells were washed with PBST 3 times and incubated with Alexa Fluor 488 goat anti-mouse antibody (ThermoFisher, Waltham, MA, USA) for 1 h at 37 °C. After intensive washing, the cells were analyzed under a fluorescence microscope (Ti-U-Nikon, Tokyo, Japan) with a video documentation system.

Western blot assay (WB) was performed to determine IAV. Briefly, the isolates were separated using 12% sodium dodecyl sulfate polyacrylamide gel electrophoresis (SDS-PAGE) and subsequently transferred onto polyvinylidene difluoride membranes. The membranes were blocked in blocking buffer containing 5% skimmed milk in Tris-buffered saline and Tween 20 (TBS-T) at 4 °C for 1 h, and incubated with the NP monoclonal antibody for 2 h at room temperature (RT). The HRP-labelled goat anti-mouse antibody (ABclonal, China) was used as the secondary antibody to incubate the membrane for 1 h at RT. After washing thrice with TBS-T, signals were visualized using the ECL chemiluminescence system. Protein bands were analyzed via Image Lab software 4.0.1.

Transmission electron microscopic (TEM) analysis was performed to observe the morphology of the SIV as reported [18]. The isolates purified by ultra-high-speed centrifugation were negatively stained with phosphotungstic acid and ammonium molybdate on 400 mesh copper mesh (Beijingzhongjingkeyi Technology Co., Ltd, China), respectively, and images were recorded on a Hitachi microscope (HT7700, Japan) with a camera.

### Whole-genome analysis of SIV

PCR was performed using segment-specific primers for eight genes (PB2, PB1, PA, HA, NP, NA, M, and NS) as reported previously [19]. The PCR products were purified and sequenced as reported previously [20]. DNA sequences were compiled and edited using Lasergene 7.1 (DNASTAR, Madison, WI, USA). Phylogenetic trees were generated by the distance-based neighbor-joining method using software MEGA 7.0 (Sinauer Associates, Inc., Sunderland, MA, USA). The reliability of the tree was assessed by bootstrap analysis with 1000 replicates.

### Infection studies in mice

To evaluate the pathogenicity of isolates in mammalian hosts, the six-week-old female BALB/c mice were purchased from the Laboratory Animal Research Center of Huazhong Agricultural University (Wuhan, China) approved by the Ethical and Welfare Committee (HZAUMO-2022-0051) and were infected intranasally with 50 µL 10^6^ 50% egg infectious dose virus (EID_50_) after anesthesia with sodium pentobarbital Additionally, 5 mice infected with 50 µL of PBS were used as negative controls. All mice were monitored for mortality and weight change over 14 days daily and euthanized immediately with 30% or greater weight loss (considered as death). The lung, trachea, spleen, ovary, and kidney collected from the infected before the agonal stage death and control mice after 14 days (humanely euthanized by injecting an overdose of intraperitoneal sodium pentobarbital) were then taken for viral titer analysis in embryonated chicken eggs by Reed and Muench.

### Histology and immunohistochemistry

The infected group before the agonal stage death and the PBS group following 14 dpi of virus isolates were humanely euthanized by injecting an overdose of intraperitoneal sodium pentobarbital and necropsied. The mice were aseptically necropsied, and the lung and trachea were collected and fixed in 10% neutral buffered formalin for HE staining [21]. Immunohistochemistry was performed to identify the position of the virus in the lungs and trachea as reported previously [22–24].

### Statistical analysis

All the statistical analyses were performed using GraphPad Prism software. Comparisons among different groups were evaluated by one-way ANOVA. Data were expressed as the mean ± the standard deviation (SD). In all cases, *p* < 0.05 was considered as statistically significant difference.

## Results

### Description of clinical cases and detection of pathogens

**Fig. 1:**
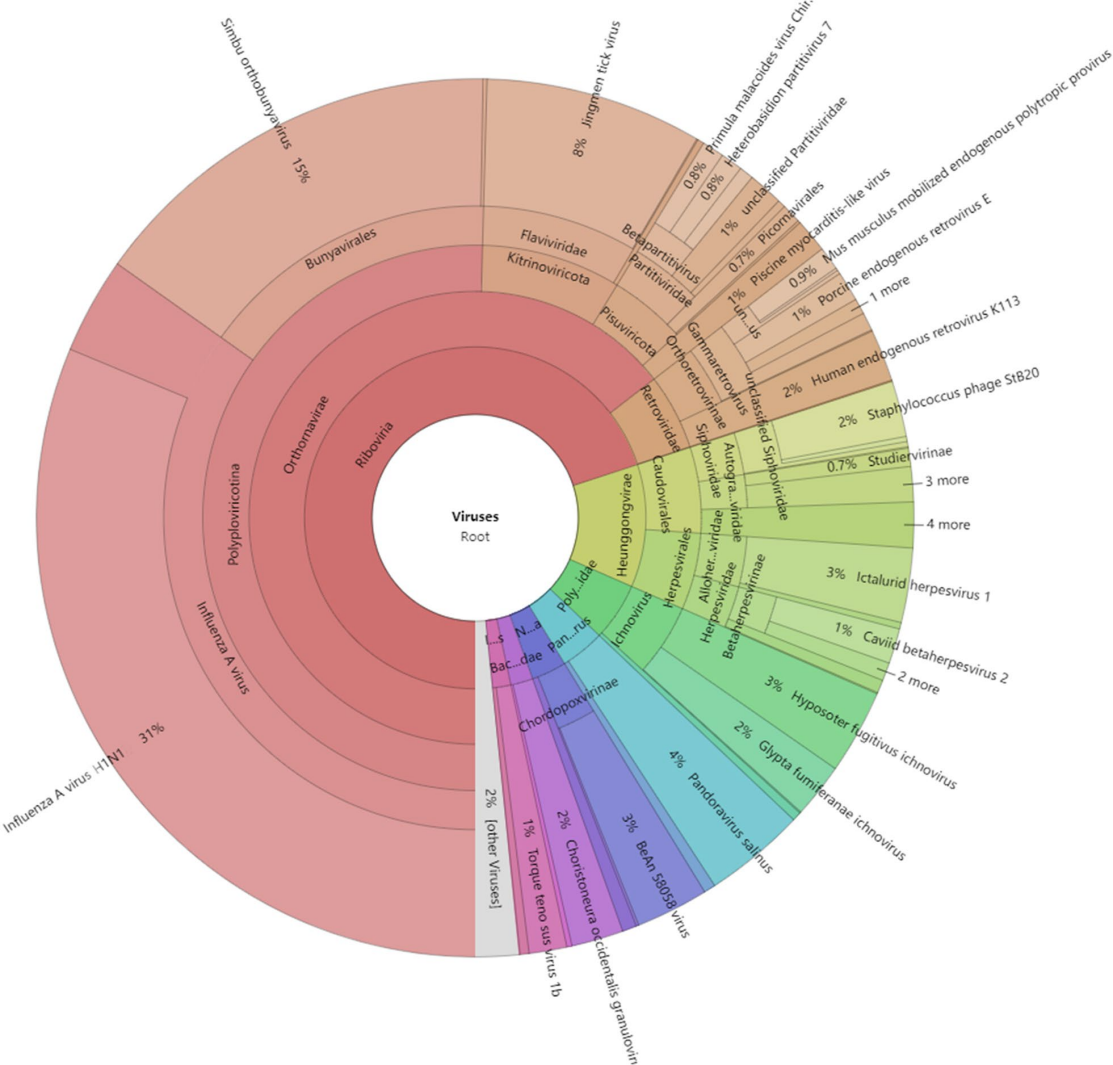
Detection of pathogens that can cause miscarriage and respiratory disease by NGS.

Pregnant pigs showed decreased appetite, fever, and intervals of 1–2 days of miscarriage (for one month), and the characterization of reproductive failure in late-gestation sows is premature farrowing of stillborn and mummified fetuses (Additional file 1: Fig. S1A). The clinical symptom of miscarriage and respiratory disease was contagious and developed sequentially from A zone to C zone to B zone, with a progressive increase in the proportion of miscarriage (Additional file 1: Fig. S1B). The major pathogens were influenza virus (31%), simbu orthobunyavirus (15%) and jingmen tick virus (8%) by NGS (Fig. 1; Table 1), while the pathogen that can cause miscarriage and respiratory disease was influenza virus. Lung samples were chosen to detect the SIV based on the partial M1 gene, and the samples were positive for SIV by RT-PCR. In addition, RT-PCR or PCR was performed to detect whether there were other viruses, no other pathogens, including classical swine fever virus (CSFV), porcine reproductive and respiratory syndrome virus (PRRSV), pseudorabies virus (PRV), Japanese encephalitis virus (JEV), porcine circovirus type 2 (PCV2), porcine circovirus type 3 (PCV3), porcine pseudorabies virus (PRV), Leptospira and Toxoplasma gondii (data not shown).

**Table 1:** The major pathogens of tissue samples from pregnant sows with miscarriage and respiratory disease by NGS.

Virus	Rates (%)
Influenza virus	31
Simbu orthobunya virus	15
Jingmen tick virus	8
Primula malacoides virus	0.8
Heterobesidion partitvirus 7	0.8
Unclassified Partitvirade	1
Picomavirales	0.7
Piscine myocarditis-Like	1
Mus musculus mobilized endogenous polytropic provirus	0.9
Porcine endogenous retrovirus E	1
Human endogenous retrovirus K113	2
Staphylococcus phage StB20	2
Studiervirinae	0.7
Ictalurid herpesvirus 1	3
Caviid betaherpesvirus	1
Hyposoter fugitivus ichnovirus	3
Glypta fumiferanae ichnovirus	2
Pandoravirus salinus	4
BeAn 58,058 virus	3
Choristoneura occidentalis granulovirus	2
Torque teno sus virus 1b	1
Other viruses	2

### Isolation, identification and phylogenetic analysis

**Fig. 2:**
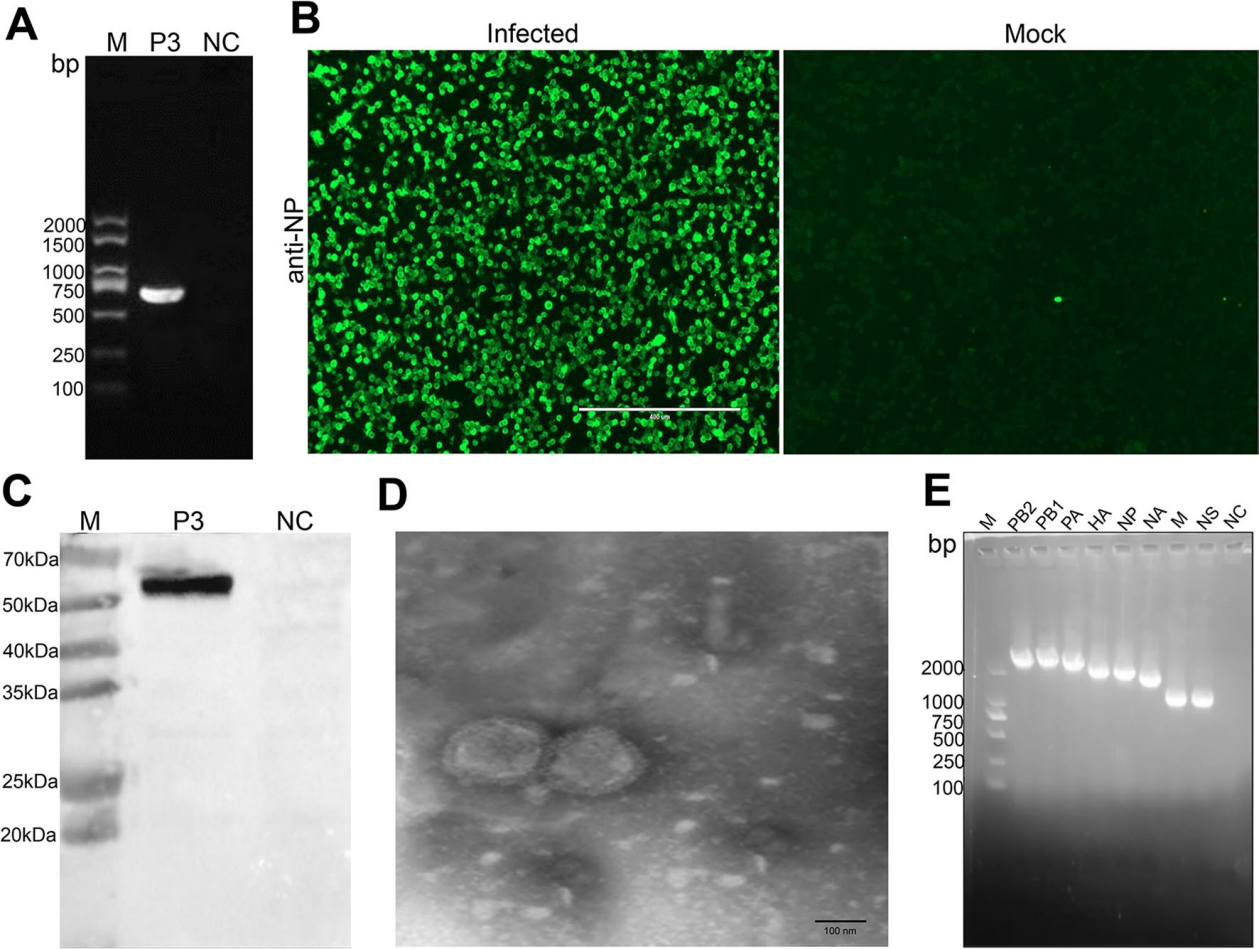
Identification of A/swine/Heilongjiang/GN/2020 strain. **A** Identification of the isolates by RT-PCR using specific primer of M gene. **B** Identification of the isolates by IFA using NP monoclonal antibody of IAV. **C** Identification of virus by WB using NP monoclonal antibody of IAV. **D** TEM shows features of an orthomyxovirus particle by negative stain (Bar = 100 nm). **E** Identification of whole-genome by RT-PCR using segment-specific primers for eight genes. P3, A/swine/Heilongjiang/GN/2020 virus was proliferated with 3 passages. NC, negative control. anti-NP. IFA, Indirect immunofluorescence assay. WB, Western blot assay. TEM: Transmission electron microscopic. PB2, PB1, PA, HA, NP, NA, M and NS were protein of IAV.

The isolates with hemagglutination activity (Additional file 1: Fig. S2) were isolated from lung samples and were then identified by RT-PCR, IFA and WB and TEM. Amplification of the isolates by specific detect-primers resulted in a target band of approximately 689 bp (Fig. 2A). The subtype of A/swine/Heilongjiang/GN/2020(H1N1) was identified as the H1 and N1 (data not shown) and was named A/swine/Heilongjiang/GN/2020(H1N1). When the NP monoclonal antibody of IAV was used as the primary antibody, the virus could be observed as green fluorescence under fluorescence microscopy by IFA and was detected with bands at ~ 55 kDa by WB (Fig. 2B, C). TEM results demonstrated characteristics of an Orthomyxovirus that enveloped spherical particles approximately 100–120 nm in diameter, and the virion surface contained dense projections (Fig. 2D). These data indicated that the SIV was isolated successfully.

**Fig. 3:**
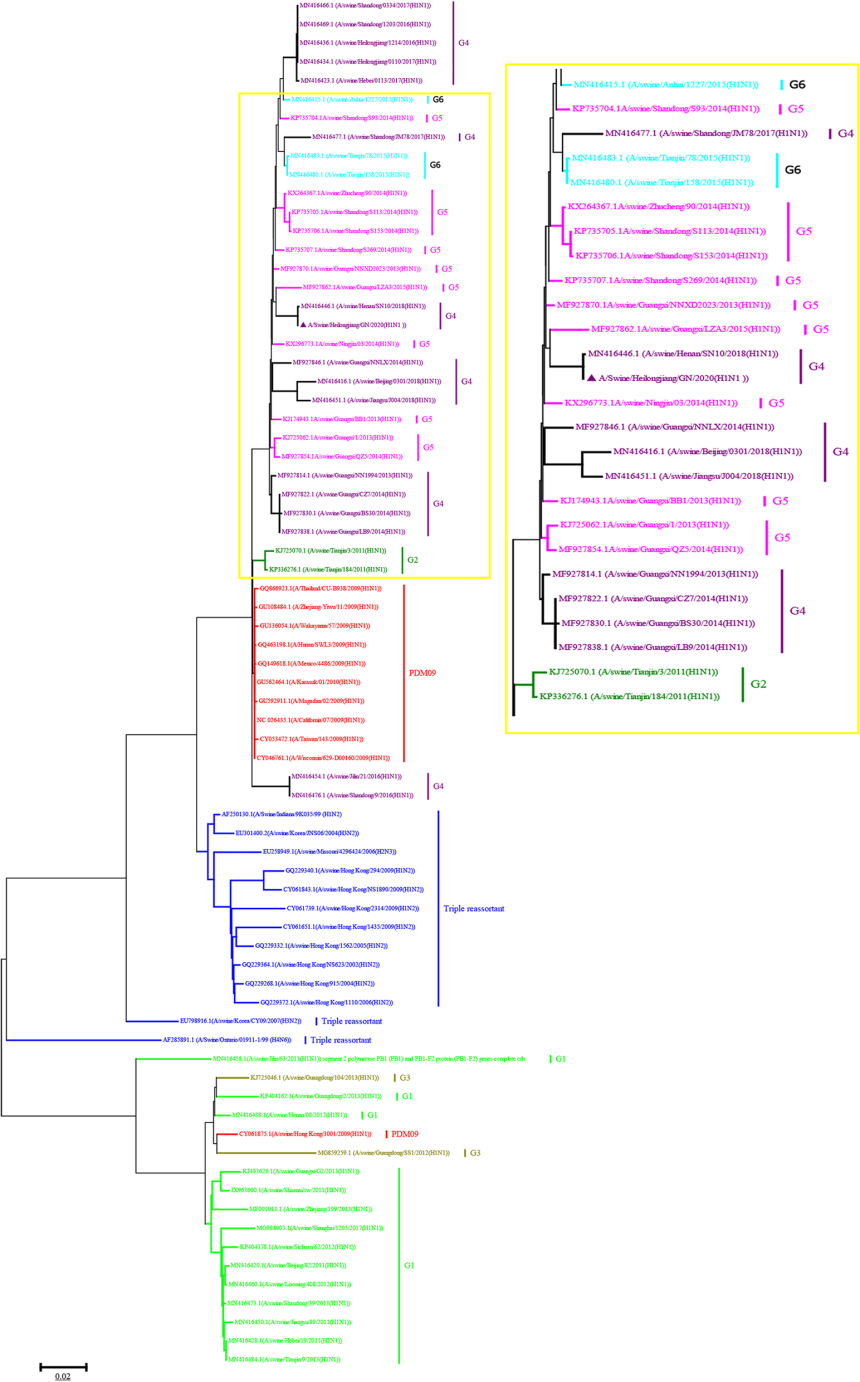
Phylogenetic analysis HA gene. The trees were constructed using the neighbor-joining method with the Maximum Composite Likelihood model and MEGA version 7.0 with 1000 bootstrap replicates. Our virus was indicated by purple triangle marker “▲”

Further genetic analysis of the virus was performed in this study. To understand the genetic evolution of the virus, the eight genes of SIV were amplified (Fig. 2E), sequenced and submitted for nucleotide BLASTn analysis of the Influenza Sequence Database in GeneBank. The eight gene segments of the virus were closely related to EA H1N1 A/swine/Henan/SN10/2018 as reported [2], which is endemic in China, with 97–100% nucleotide sequence homology (Fig. 3, Additional file 1: Fig. S3 and Table S2). The PB2, PB1, PA, NP, and M genes of the isolate are also confirmed to have high homology with pdm09-H1N1 A/Mexico/4486/2009, which appeared in Mexico in 2009, with 97.3–98% nucleotide sequence homology. The HA and NA genes of this virus are closely related to EA-H1N1 A/swine/Beijing/82/2011 in China, with 97.3–97.4% nucleotide sequence homology. The NS gene of the virus is closely related to TP-H1N2 A/swine/Hong Kong/294/2009 from China with 93.8% homology. These data demonstrated the HA and NA genes of this virus belong to the EA lineage. The other genes (PB2, PB1, PA, NP, M) belong to the pdm09 lineage, while the NS gene belong to the TP lineage. The results suggested that A/swine/Heilongjiang/GN/2020(H1N1) was confirmed as the EA-H1N1 genotype of SIV. The accession numbers for each of the eight segments of A/swine/Heilongjiang/GN are OP647087 to OP647094(segment 1 (PB2): OP647087; segment 2 (PB1): OP647088; segment 3 (PA): OP647089; segment 4 (HA): OP647090; segment 5 (NP): OP647091; segment 6 (NA): OP647092; segment 7 (M1 and M2): OP647093; segment 8 (NS1 and Nep): OP647094;), respectively.

### Molecular characterization of HA1 genes

**Fig. 4:**
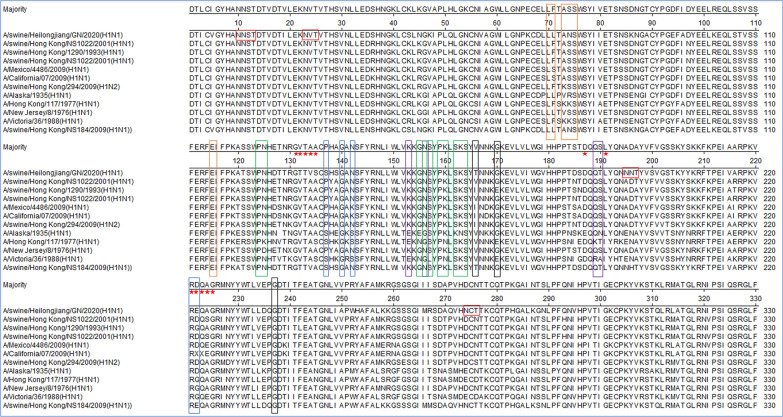
Molecular analysis of HA1 amino acid sequences of the isolates and reference strains. Potential glycosylation sites were marked with red box. Previously defined antigenic sites were indicated: Site Sa (green box), site Sb (purple box), site Ca1 (black shade), site Ca2 (blue box), Cb (orange box). The pentagram represents the receptor-binding sites. A/HongKong/1290/1993(H1N1) and A/swine/Hong Kong/NS1022/2001(H1N1) are all from the classical swine lineage. A/California/04/2009(H1N1) and A/Mexico/4486/2009(H1N1) are from the pandemic H1N1/2009 sublineage of classical swine lineage. A/HongKong/294/2009(H1N2) is from the triple reassortant sublineage of classical swine lineage. A/Alaska/1935(H1N1), A/New Jersey/1976(H1N1), A/Hong Kong/117/1977(H1N1) and A/Victoria/36/1988(H1N1) are all from the human lineage. A/swine/Hong Kong/NS184/2009(H1N1) is from the avian lineage

To understand whether the signature amino acids of HA1 genes associated with virulence and host adaptation had changed, molecular characterization of A/swine/Heilongjiang/GN/2020 and the reference viruses of H1N1 from different lineages were analyzed. As shown in Fig. 4, the HA gene of A/swine/Heilongjiang/GN/2020 encoded 566 amino acids and contained the motif PSIQSR/GLF at the cleavage site between HA1 and HA2, which is consistent with the characteristics of low pathogenic IAV [25]. The virus had five potential glycosylation sites (N-X-S/T) at positions 10 (NNS), 11 (NST), 23 (NVT), 195 (NNT), and 274(NCT). The binding preference of HA to host SAα2,6Gal receptor is a critical determinant for the cross-species transmission of IAVs to humans [26]. As shown in Fig. 4, the amino acid residues at positions 131–135, 187, 191, and 221–225 are well-established amino acid positions related to the receptor specificity of IAV [9]; Amino acid residues at the receptor-binding pocket of HA1 (position Q223 and G225) retained configurations (SAα2,6Gal) in A/swine/Heilongjiang/GN/2020, predicting that it had an affinity for mammalian cell-surface receptors; The virus and the reference viruses had the same amino acid residues at G131, T133, and R221; Though varying degrees of mutation at positions 132, 134, 135, and 222 occurred, the virus had the same amino acids with the reference influenza virus A/swine/Hong Kong/NS184/2009(H1N1) of avian lineage; The antigenic determinant domains (Sa, Sb, Ca1, Ca2, and Cb) were found to be involved in antibody binding [27, 28], and the major antigenic determinant domains of the virus were similar to the reference H1N1 influenza viruses of swine and avian. In the antigenic site of Ca1, the mutation T204S was found in the virus. These data suggest that the virus could adapt the host to infect avian species, swine and humans by receptor-binding, and the antibody produced by seasonal IAV H1N1 elicited limited cross-reactivity against the virus because of the mutation of antigenic determinant domains, as reported previously [2].

### Pathogenicity in mice

**Fig. 5:**
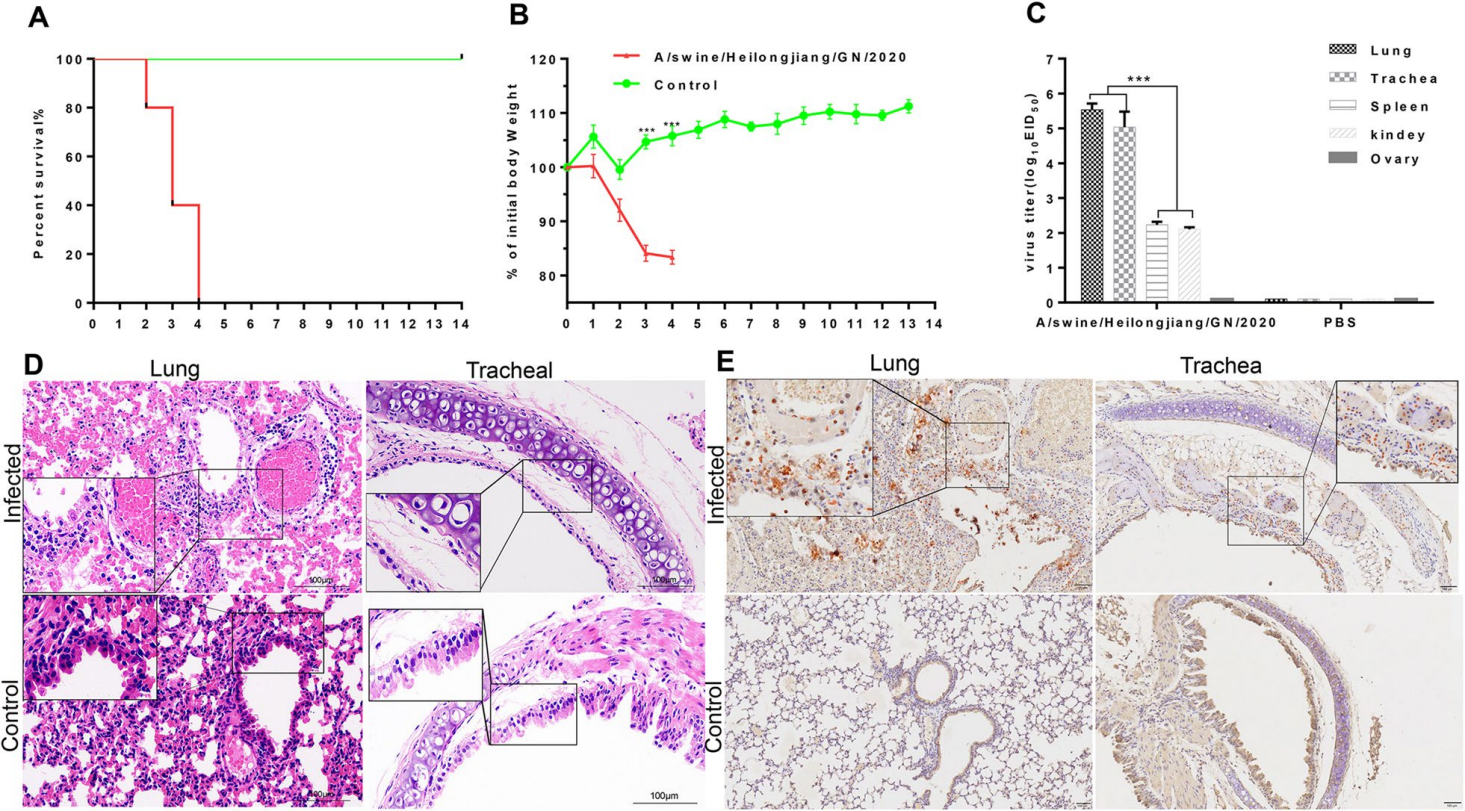
Pathogenicity of the isolates in mice. **A** Lethality of A/swine/Heilongjiang/GN/2020 in mice. **B** Weight change of mice during the 14 dpi. **C** Viral load of tissues in infected mice, and their organs were collected for virus titration. **D–E** Histopathological examination of lung and trachea tissues. Representative images of hematoxylin-and-eosin staining and immunohistochemistry from mice infected A/swine/Heilongjiang/GN/2020 before the agonal stage death. Data are represented as the mean ± SD (n = 3). *, *p* < 0.05. **, *p* < 0.01. ***, *p* < 0.001 (ANOVA)

In general, mice serve as a useful animal model system for evaluating the virulence of influenza viruses to humans [29]. In our study, BALB/c mice were infected with 10^6^ EID_50_ of A/swine/Heilongjiang/GN/2020 and were monitored for 14 days for signs of illness, weight loss, and mortality. Their lung, trachea, spleen, ovary, and kidney were collected for virus titration. The mice infected with the A/swine/Heilongjiang/2020 virus displayed erect hair, chills, reduced appetite, and severe weight loss (decreased by 16.62%) starting from 1 dpi, and all had died by 4 dpi (Fig. 5A, B). The virus to the high titer of 5.6 log10 EID_50_/mL in the lungs and to the mean titers of 5 log10 EID_50_/mL, 2.2 log10 EID_50_/mL, 2.1 log10 EID_50_/mL and 0 EID_50_/mL in the trachea, spleen, kidney, and ovary, respectively, which indicated the level of virus titer in the extrapulmonary organs (spleen and kidney) was significantly lower than that in respiratory tracts (lung and trachea) (*p* < 0.001) (Fig. 5C). The mice in the control group did not show any clinical symptoms and gained weight (increased by 11.24%) over the course of the observation period, with no virus detected in the lung, trachea, spleen, ovary, or kidney at 14 dpi. The body weight of mice infected was significantly lower than that of the control mice from 1 dpi to 4 dpi (*p* < 0.01) (Fig. 5B). Overall, these data suggest that A/swine/Heilongjiang/2020 had high pathogenicity in mice.

### Histopathological to lungs and trachea

To correlate virulence with pathogenicity, lung and trachea tissues from the infected mice were collected before the agonal stage death for pathological and immunohistochemistry assessment [30]. Mice infected with the virus developed severe histopathological damage in both the lungs and trachea. As shown in Fig. 5D, the histopathological changes showed that the alveolar structure was severely damaged, and a large number of epithelial cells were necrotic and exfoliated. There were a large number of exfoliated epithelial cells and a small number of neutrophils, lymphocytes, and macrophages that engulfed necrotic cell debris in the alveolar cavity. The ciliated epithelium of the tracheal mucosa was severely atrophied, from pseudostratified to monolayered, the submucosa showed edema with congestion, the chondrocytes were basophilic, and the adventitia was shed. To identify the position of the virus in lung and trachea, the NP monoclonal antibody was used as the primary antibody in immunohistochemistry. Immunostaining of lung and trachea sections revealed prominent expression on alveolar cells and alveolar epithelial cells, including damaged, desquamated cells within the alveolar space, while viral protein expression was minimally detectable on the cells of blood vessels (Fig. 5E, left panels). The expression of the viral protein on ciliated epithelial cells in the trachea, including columnar epithelial cells and submucosal layers, is higher than that in the non-ciliated cuboidal epithelium (Fig. 5E, right panels).

## Discussion

Swine influenza was first recognized as a disease of pigs during the human pandemic in 1918 [31]. Pigs are an important host in IAV ecology, since they are susceptible to infection with avian, porcine, and human IAV. Avian/human, human/swine, and TP reassortant IAV have been isolated from pigs worldwide [32]. The sequences of EA H1N1 SIVs from China between 2001 and 2020, including the 2009 pandemic H1N1 (A(H1N1) pdm09), and triple reassortant H1N2 (TP H1N2) influenza viruses, with SIVs generating 11 genotypes [1, 2]. Presently, there are various subtypes of swine influenza virus in China. H1N1, H3N2 and H1N2 were the predominant genetic subtypes of the virus in pigs, and H4N8, H5N1, H6N6, H7N9, H9N2 and H10N8 SIV also were directly transmitted from to the pigs since 2009 to 2021 [33–37]. In our study, a H1N1 virus (A/swine/Heilongjiang/GN/2020(H1N1)) was isolated from pigs with miscarriage and respiratory disease. Homology and phylogenetic analyses of whole-genome results demonstrated that the PB2, PB1, PA, NP, and M genes of the virus are confirmed to have high homology with pdm09-H1N1 viruses nucleotide sequence, and the HA and NA genes of the virus are closely related to the nucleotide sequence homology of EA-H1N1 viruses, while the NS gene of the virus is closely related to TP viruses from China (Additional file 1: Fig. S3). These data indicated that the origin of eight gene fragments of the virus is consistent with EA-H1N1 SIV as reported previously [2], so A/swine/Heilongjiang/GN/2020 belongs to the EA-H1N1 genotype.

The deduced amino acid sequences of A/swine/Heilongjiang/GN/2020 and the reference viruses of H1N1 from different lineages were analyzed to determine whether the signature amino acids of HA1 genes associated with virulence and host adaptation had changed [38]. The HA gene of A/swine/Heilongjiang/GN/2020 contained the motif PSIQSR/GLF at the cleavage site between HA1 and HA2 consistent with the characteristics of low pathogenic IAV as reported previously (Fig. 4) [7, 25]. The glycosylation positions 195 (NNT) and 274 (NCT) of A/swine/Heilongjiang/GN/2020 are inconsistent with that of the CS lineage (Fig. 4), while their changes might enable the influenza virus to escape the host immune system as reported previously [39, 40].

The binding preference of HA to the host SAα2,6Gal receptor is a critical determinant for the cross-species transmission of IAVs to humans [26]. The HAs of human IAVs preferentially bind to the SAα2,6Gal receptor, while avian IAVs prefer the SAα2,3Gal receptor [41]. EA-H1N1 replicate similarly to pdm/09-H1N1, both preferentially bind human-like SAα2,6Gal receptor and replicate efficiently in human airway epithelial cells, as reported [2]. Amino acid residues at the receptor-binding position of HA1 (positions Q223 and G225) retained SAα2,6Gal receptor and SAα2,3Gal (G131-A135) in the virus, predicting that it had an affinity for mammalian cell-surface receptors that could adapt host to infect avian species, swine and humans by receptor-binding. The antigenic determinant domains (Sa, Sb, Ca1, Ca2, and Cb) were found to be involved in antibody binding [27, 28], and the major antigenic determinant domains of A/swine/Heilongjiang/GN/2020 were similar to the reference influenza. However, in the antigenic site of Ca1, the mutation T204S was found in the virus (Fig. 4), and the antibody produced by seasonal IAV H1N1 elicited limited cross-reactivity against the virus because of mutation of the antigenic determinant domains [7].

Previous studies showed that EA-H1N1 viruses and their cases displayed increased pathogenicity [11]. A recent study found that EA H1N1 viruses formed eight different genotypes through reassortment with viruses of other lineages circulating in humans and pigs, two of these genotypes (G4 and G5) were widely distributed in pigs as reported in 22 provinces in China between October 2013 and December 2019 [14]. In our study, the respiratory disease in mice infected with the EA-H1N1(A/swine/Heilongjiang/GN/2020) resulted in greater weight loss and was lethal, which is similar to the outcome of mice infected with TP A/Ohio/2/07 (OH/2) virus [42]. More interestingly, the replication of A/swine/Heilongjiang/GN/2020 was not restricted to respiratory tract tissues but could be systemically spread in mice. The A/swine/Heilongjiang/GN/2020 showed effective replication in lung, trachea, spleen and kidney of mice (Fig. 5C), which is consistent with the lethal and systemic transmission of EA-H1N1 viruses in mice [43], and was inconsistent with pdm09 virus not causing lethal or exhibit extrapulmonary virus spread [44]. Yang et al. reported that the EAH1N1 SIVs are preferentially bound to human-type receptors, which suggests that is needed to prevent the efficient transmission of EAH1N1 SIVs to humans as reported previously [45]. Meng et al. found that EA H1N1 viruses obtained pathogenic in mice characteristics by accumulating mutations in its acidic polymerase (PA) gene that is predominant in the pdm09-H1N viruses [46]. Wang et al. found that a single-amino-acid substitution of glycine (G) for glutamic acid (E) at position 225 (E225G) in the HA1 protein completely abolished the respiratory droplet transmission of GX/18, whereas the substitution in the same position (G225E) in HA1 enabled HLJ/27 to transmit in guinea pigs [47]. In our study, the PA gene of A/swine/Heilongjiang/GN/2020 belongs to the pdm09 lineage (Additional file 1: Figure S3C) and the amino acid residues at the receptor-binding pocket of HA1 (position Q223 and G225) retained configurations (SAα2,6Gal) (Fig. 4), which suggests that that the virus could be effectively transmitted to humans through mice. The characterization of reproductive failure in late-gestation sows is premature farrowing of stillborn and mummified fetuses, and the pathogen of which only was SIV and no other pathogens by NGS and PCR analysis. So, is miscarriage associated with significant proliferation of influenza virus in the ovary or uterus? We attempted to determine whether the virus could be isolated from the ovary of mice infected with A/swine/Heilongjiang/2020, but it was unfortunately unsuccessful (Fig. 5C). Pregnant sows have an increased risk of respiratory complications and reproductive failure related to influenza as reported previously [48], which may also be responsible for uterine contractions constituting a threat of miscarriage or premature labor. Nonetheless, the mechanism of reproductive failure that EA-H1N1caused reproductive failure in late-gestation sows remains to be further explored in pigs. Histopathological and immunohistochemistry examinations showed that the virus could cause severe damage of respiratory tracts and proliferated effectively lungs and trachea (Fig. 5D, E). These results found that the EA H1N1 virus (A/swine/Heilongjiang/GN/2020) was highly pathogenic to mice and could be systemic spread in mice.

EA-H1N1 viruses have been a growing problem in pig farms since 2014, inevitably increasing their exposure to humans [49]. Two human cases of EA-lH1N1 infection have been reported in 2016 and 2019 in China [49, 50], and genetic analysis indicated that the two cases were caused by the EA-H1N1 virus [2]. The two patients’ neighbors who reared pigs, suggesting that the EA-H1N1 virus could transmit from swine to humans [49–51]. Thus, virological surveillance of EA-H1N1 is necessary for swine and human populations, which has fundamental implications for human public health.

## Conclusion

A/swine/Heilongjiang/GN/2020 was isolated from pregnant sows with miscarriage and respiratory disease in Heilongjiang province, China. Homology and phylogenetic analysis showed that the virus is most closely related to A/swine/Henan/SN/10/2018 and belonged to A-H1N1. The virus retained configurations (SAα2,6Gal) in HA1 (position Q223 and G225), predicting that it had an affinity for mammalian by receptor-binding. Pathogenicity in mice showed that A/swine/Heilongjiang/GN/2020 could cause lethal or exhibit extrapulmonary virus spread and cause severe damage to respiratory tracts effectively proliferating in lungs and tracheas.

## Supplementary Information

**Additional file 1**: **Table S1** The primers were used in our study. **Table S2** The nucleotide homology between the A/swine/Heilongjiang/GN/2020 and A/swine/Henan/SN10/2018 virus, when analyzing each gene fragment. **Fig.** **S1** Description of the clinical case. (A) The clinical symptom of miscarriage in pregnant sows. (B) Epidemiology of miscarriage in pregnant sows. **Fig. S2** Identification of hemagglutination activity of the isolate virus. The virus P3-P5 generations were diluted at a 2-fold ratio and 30uL of virus was then added to 30uL of 1% chicken red blood cells to determine hemagglutination. **Fig. S3** Phylogenetic analysis of the PB2 (A), PB1 (B), PA (C), NP (D), NA(E), M (F), and NS (G)genes. The trees were constructed by using the neighbor-joining method with the Maximum Composite Likelihood model and MEGA version 7.0 with 1,000 bootstrap replicates. The virus isolated in this study was indicated by purple triangle marker “▲”. **Figure S4** The 50% mice lethal dose (MLD50) of A/swine/Heilongjiang/2020. The six-week-old female BALB/c mice were infected intranasally with 50 uL 101 to106 50% egg infectious dose virus (EID50) to detect MLD50.

## Data Availability

The data that support the findings of this study are available from the corresponding author upon reasonable request.

## Abbreviations

NGS: Next-generation sequencing
SIV: Swine influenza viruses
EA-H1N1: Eurasian avian-like H1N1
IAV: Influenza A viruses
TRI: TRIzol reagent
pdm09-H1N1: Pandemic 2009 H1N1 viruses
TP: Human/avian/swine triple-reassortant viruses
EA H1N1: Eurasian avian-like H1N1 viruses
PB2: Polymerase PB2 protein of IAV
PB1: Polymerase PB1 protein of IAV
PA: Polymerase PA protein of IAV
HA: Hemagglutinin protein of IAV
NP: Nucleocapsid protein of IAV
NA: Neuraminidase protein of IAV
NS: Nonstructural protein protein of IAV
M: Matrix protein of IAV
MDCK: Madin-Darby canine kidney cells
TCID_50_: 50% tissue culture infective dose
RT‑PCR: Reverse‑transcription polymerase chain reaction
IFA: Indirect immunofluorescence assay
WB: Western blot assay
TEM: Transmission electron microscopic
SDS-PAGE: Sodium dodecyl sulfate polyacrylamide gel electrophoresis
RT: Room temperature
EID_50_: 50% egg infectious dose virus
HE: Hematoxylin-and-eosin
dpi: Days post-infection

## References

[1] Feng Z, Zhu W, Yang L, Liu J, Zhou L, Wang D, Shu Y (2021). Epidemiology and genotypic diversity of Eurasian Avian-Like H1N1 Swine Influenza Viruses in China. Virol Sin.

[2] Sun H, Xiao Y, Liu J, Wang D, Li F, Wang C (2020). Prevalent eurasian avian-like H1N1 swine influenza virus with 2009 pandemic viral genes facilitating human infection. Proc Natl Acad Sci U S A.

[3] Abdelwhab EM, Hafez HM (2011). An overview of the epidemic of highly pathogenic H5N1 avian influenza virus in Egypt: epidemiology and control challenges. Epidemiol Infect.

[4] Wille M, Holmes EC. The ecology and evolution of Influenza viruses. Cold Spring Harb Perspect Med. 2020; 10:https://www.ncbi.nlm.nih.gov/pubmed/31871237. 10.1101/cshperspect.a038489 PMC7328453 31871237

[5] Shi Y, Wu Y, Zhang W, Qi J, Gao GF (2014). Enabling the ‘host jump’: structural determinants of receptor-binding specificity in influenza A viruses. Nat Rev Microbiol..

[6] Imai M, Kawaoka Y (2012). The role of receptor binding specificity in interspecies transmission of influenza viruses. Curr Opin Virol.

[7] Song Y, Zhang Y, Zhang B, Chen L, Zhang M, Wang J, Jiang Y, Yang C, Jiang T. Identification, genetic analysis, and pathogenicity of classical swine H1N1 and human-swine reassortant H1N1 influenza viruses from Pigs in China. Viruses. 2020; 12:https://www.ncbi.nlm.nih.gov/pubmed/31906591. 10.3390/v12010055 PMC7019673 31906591

[8] Peiris JS, Poon LL, Guan Y (2009). Emergence of a novel swine-origin influenza a virus (S-OIV) H1N1 virus in humans. J Clin Virol.

[9] Vijaykrishna D, Smith GJ, Pybus OG, Zhu H, Bhatt S, Poon LL (2011). Long-term evolution and transmission dynamics of swine influenza a virus. Nature.

[10] Pulit-Penaloza JA, Belser JA, Tumpey TM, Maines TR (2019). Mammalian pathogenicity and transmissibility of a reassortant eurasian avian-like a(H1N1v) influenza virus associated with human infection in China (2015). Virology..

[11] Wang G, Dos Anjos Borges LG, Stadlbauer D, Ramos I, Bermudez Gonzalez MC, He J (2019). Characterization of swine-origin H1N1 canine influenza viruses. Emerg Microbes Infect.

[12] Cao Z, Zeng W, Hao X, Huang J, Cai M, Zhou P, Zhang G (2019). Continuous evolution of influenza A viruses of swine from 2013 to 2015 in Guangdong, China. PLoS ONE.

[13] Matranga CB, Andersen KG, Winnicki S, Busby M, Gladden AD, Tewhey R (2014). Enhanced methods for unbiased deep sequencing of Lassa and Ebola RNA viruses from clinical and biological samples. Genome Biol.

[14] Meng F, Chen Y, Song Z, Zhong Q, Zhang Y, Qiao C, et al: Continued evolution of the eurasian avian-like H1N1 swine influenza viruses in China. Sci China Life Sci. 2022; https://www.ncbi.nlm.nih.gov/pubmed/36219302. 10.1007/s11427-022-2208-0 36219302

[15] Hoffmann E, Webster RG (2000). Unidirectional RNA polymerase I-polymerase II transcription system for the generation of influenza a virus from eight plasmids. J Gen Virol.

[16] Tsukamoto K, Ashizawa H, Nakanishi K, Kaji N, Suzuki K, Okamatsu M, Yamaguchi S, Mase M (2008). Subtyping of avian influenza viruses H1 to H15 on the basis of hemagglutinin genes by PCR assay and molecular determination of pathogenic potential. J Clin Microbiol.

[17] Tsukamoto K, Ashizawa T, Nakanishi K, Kaji N, Suzuki K, Shishido M, Okamatsu M, Mase M (2009). Use of reverse transcriptase PCR to subtype N1 to N9 neuraminidase genes of avian influenza viruses. J Clin Microbiol.

[18] Hause BM, Ducatez M, Collin EA, Ran Z, Liu R, Sheng Z (2013). Isolation of a novel swine influenza virus from Oklahoma in 2011 which is distantly related to human influenza C viruses. PLoS Pathog.

[19] Hoffmann E, Stech J, Guan Y, Webster RG, Perez DR (2001). Universal primer set for the full-length amplification of all influenza a viruses. Arch Virol.

[20] Zhang H, Chen P, Hao G, Liu W, Chen H, Qian P, Li X. Comparison of the pathogenicity of two different branches of Senecavirus a strain in China. Pathogens. 2020; 9:https://www.ncbi.nlm.nih.gov/pubmed/31906571. 10.3390/pathogens9010039 PMC7168630 31906571

[21] Li J, Fang K, Rong Z, Li X, Ren X, Ma H, Chen H, Li X, Qian P. Comparison of gE/gI- and TK/gE/gI-Gene-deleted pseudorabies virus vaccines mediated by CRISPR/Cas9 and Cre/Lox Systems. Viruses. 2020; 12:https://www.ncbi.nlm.nih.gov/pubmed/32230737. 10.3390/v12040369 PMC7232343 32230737

[22] Pantin-Jackwood MJ (2020). Immunohistochemical staining of Influenza Virus in tissues. Methods Mol Biol.

[23] Zhang H, Zhou P, Wei Y, Yue H, Wang Y, Hu M (2020). Histopathologic changes and SARS-CoV-2 immunostaining in the lung of a patient with COVID-19. Ann Intern Med.

[24] Thongratsakul S, Suzuki Y, Hiramatsu H, Sakpuaram T, Sirinarumitr T, Poolkhet C, Moonjit P, Yodsheewan R, Songserm T (2010). Avian and human influenza a virus receptors in trachea and lung of animals. Asian Pac J Allergy Immunol.

[25] Dupre G, Hoede C, Figueroa T, Bessiere P, Bertagnoli S, Ducatez M, Gaspin C, Volmer R (2021). Phylodynamic study of the conserved RNA structure encompassing the hemagglutinin cleavage site encoding region of H5 and H7 low pathogenic avian influenza viruses. Virus Evol.

[26] Matrosovich M, Tuzikov A, Bovin N, Gambaryan A, Klimov A, Castrucci MR, Donatelli I, Kawaoka Y (2000). Early alterations of the receptor-binding properties of H1, H2, and H3 avian influenza virus hemagglutinins after their introduction into mammals. J Virol.

[27] Caton AJ, Brownlee GG, Yewdell JW, Gerhard W (1982). The antigenic structure of the influenza virus A/PR/8/34 hemagglutinin (H1 subtype). Cell.

[28] Wiley DC, Wilson IA, Skehel JJ (1981). Structural identification of the antibody-binding sites of Hong Kong influenza haemagglutinin and their involvement in antigenic variation. Nature.

[29] Mifsud EJ, Tai CM, Hurt AC (2018). Animal models used to assess influenza antivirals. Expert Opin Drug Discov.

[30] Li M, Guo P, Chen C, Feng H, Zhang W, Gu C, Wen G, Rao VB, Tao P (2021). Bacteriophage T4 vaccine platform for next-generation influenza vaccine development. Front Immunol.

[31] Ma W (2020). Swine influenza virus: current status and challenge. Virus Res.

[32] Thacker E, Janke B (2008). Swine influenza virus: zoonotic potential and vaccination strategies for the control of avian and swine influenzas. J Infect Dis.

[33] Kong W, Ye J, Guan S, Liu J, Pu J (2014). Epidemic status of swine influenza virus in china. Indian J Microbiol.

[34] Gu M, Xu L, Wang X, Liu X (2017). Current situation of H9N2 subtype avian influenza in China. Vet Res.

[35] Zhang G, Kong W, Qi W, Long LP, Cao Z, Huang L (2011). Identification of an H6N6 swine influenza virus in southern China. Infect Genet Evol.

[36] Zhou P, Zhu W, Gu H, Fu X, Wang L, Zheng Y (2014). Avian influenza H9N2 seroprevalence among swine farm residents in China. J Med Virol.

[37] Fu X, Huang Y, Fang B, Liu Y, Cai M, Zhong R, Huang J, Wenbao Q, Tian Y, Zhang G (2020). Evidence of H10N8 influenza virus infection among swine in southern China and its infectivity and transmissibility in swine. Emerg Microbes Infect.

[38] Kordyukova L (2017). Structural and functional specificity of Influenza virus haemagglutinin and paramyxovirus fusion protein anchoring peptides. Virus Res.

[39] Gao R, Gu M, Shi L, Liu K, Li X, Wang X (2021). N-linked glycosylation at site 158 of the HA protein of H5N6 highly pathogenic avian influenza virus is important for viral biological properties and host immune responses. Vet Res.

[40] Kim JI, Lee I, Park S, Hwang MW, Bae JY, Lee S, Heo J, Park MS, Garcia-Sastre A, Park MS (2013). Genetic requirement for hemagglutinin glycosylation and its implications for influenza A H1N1 virus evolution. J Virol.

[41] Liu K, Gu M, Hu S, Gao R, Li J, Shi L (2018). Genetic and biological characterization of three poultry-origin H5N6 avian influenza viruses with all internal genes from genotype S H9N2 viruses. Arch Virol.

[42] Pulit-Penaloza JA, Pappas C, Belser JA, Sun X, Brock N, Zeng H, Tumpey TM, Maines TR. Comparative in Vitro and in vivo analysis of H1N1 and H1N2 variant influenza viruses isolated from humans between 2011 and 2016. J Virol. 2018; 92:https://www.ncbi.nlm.nih.gov/pubmed/30158292. 10.1128/JVI.01444-18 PMC6206486 30158292

[43] Zhu W, Zhang H, Xiang X, Zhong L, Yang L, Guo J (2016). Reassortant Eurasian Avian-Like Influenza A(H1N1) virus from a severely ill child, Hunan Province, China, 2015. Emerg Infect Dis.

[44] Belser JA, Wadford DA, Pappas C, Gustin KM, Maines TR, Pearce MB, Zeng H, Swayne DE, Pantin-Jackwood M, Katz JM, Tumpey TM (2010). Pathogenesis of pandemic influenza A (H1N1) and triple-reassortant swine influenza A (H1) viruses in mice. J Virol.

[45] Yang H, Chen Y, Qiao C, He X, Zhou H, Sun Y (2016). Prevalence, genetics, and transmissibility in ferrets of eurasian avian-like H1N1 swine influenza viruses. Proc Natl Acad Sci U S A.

[46] Meng F, Yang H, Qu Z, Chen Y, Zhang Y, Zhang Y, Liu L, Zeng X, Li C, Kawaoka Y, Chen H (2022). A eurasian avian-like H1N1 swine influenza reassortant virus became pathogenic and highly transmissible due to mutations in its PA gene. Proc Natl Acad Sci U S A.

[47] Wang Z, Yang H, Chen Y, Tao S, Liu L, Kong H, Ma S, Meng F, Suzuki Y, Qiao C, Chen H. A single-amino-acid substitution at position 225 in Hemagglutinin alters the transmissibility of Eurasian Avian-Like H1N1 Swine Influenza Virus in Guinea Pigs. J Virol. 2017; 91:https://www.ncbi.nlm.nih.gov/pubmed/28814518. 10.1128/JVI.00800-17 PMC5640871 28814518

[48] Anselem O, Floret D, Tsatsaris V, Goffinet F, Launay O (2013). Influenza infection and pregnancy. Presse Med.

[49] Rewar S, Mirdha D, Rewar P (2015). Treatment and prevention of pandemic H1N1 Influenza. Ann Glob Health.

[50] Xie JF, Zhang YH, Zhao L, Xiu WQ, Chen HB, Lin Q, Weng YW, Zheng KC (2018). Emergence of Eurasian Avian-Like Swine Influenza A (H1N1) virus from an adult case in Fujian Province, China. Virol Sin.

[51] Gu M, Chen K, Ge Z, Jiao J, Cai T, Liu S, Wang X, Jiao X, Peng D, Liu X (2022). Zoonotic threat of G4 genotype eurasian avian-like Swine Influenza A(H1N1) viruses, China, 2020. Emerg Infect Dis.

