# Supplementary material for: Genetic characterization and pathogenicity of a Eurasian avian-like H1N1 swine influenza reassortant virus
Source: Virol J. 2022 Dec 2;19:205. doi: 10.1186/s12985-022-01936-6 (PMC9716174; doi:10.1186/s12985-022-01936-6)

Supplementary Material

**Table S1** The primers were used in our study.

| **gene** | **prime** | **sequence (5’-3’)** | **Size (bp)** |
| --- | --- | --- | --- |
| PB2 | PB2-F | AGCAAAAGCAGGTCAAATATATTC | 2340 |
|  | PB2-R | TAGTAGAAACAAGGTCGTTTTAAAC |  |
| PB1 | PB1-F | AGCAAAAGCAGGCAAACCA | 2342 |
|  | PB1-R | TAGTAGAAACAAGGCATTTTTTCAT |  |
| PA | PA -F | AGCAAAAGCAGGTACTGATC | 2234 |
|  | PA -R | TAGTAGAAACAAGGTACTTTTTTGG |  |
| HA | HA-F | AGCAAAAGCAGGGGAAAACAA | 1769 |
|  | HA-R | TAGTAGAAACAAGGGTGTTTTTTTC |  |
| NP | NP -F | AGCAAAAGCAGGGTAGATAA | 1566 |
|  | NP -R | TAGTAGAAACAAGGGTATTTTTCCT |  |
| NA | NA-F | AGCAAAAGCAGGAGTTTAAAAT | 1403 |
|  | NA-R | TAGTAGAAACAAGGAGTTTTTTGAAC |  |
| M | M -F | AGGAAAAGCAGGTAGATATTTAAAG | 1028 |
|  | M -R | TAGTAGAAACAAGGTAGTTTTTTACTC |  |
| NS | NS -F | GCAAAAGCAGGGTGACAAA | 889 |
|  | NS -R | TAGTAGAAACAAGGGTGTTTTTTATC |  |
| M1 | detect-F | AACGTACGTTCTTTCTATCATCC | 689 |
|  | detect-F | GCAAATTTTCAAGAAGGTCATCTT |  |

**Table S2** The nucleotide homology between the A/swine/Heilongjiang/GN/2020 and A/swine/Henan/SN10/2018 virus, when analyzing each gene fragment.

| **virus** | **gene** | **Virus with similarity** | **Homology (%)** |
| --- | --- | --- | --- |
| A/swine/Heilongjiang/GN/2020 | PB2 | A/swine/Henan/SN10/2018 | 99.7% |
|  | PB1 | A/swine/Henan/SN10/2018 | 100% |
|  | PA | A/swine/Henan/SN10/2018 | 100% |
|  | HA | A/swine/Henan/SN10/2018 | 99.9% |
|  | NP | A/swine/Henan/SN10/2018 | 99.9% |
|  | NA | A/swine/Henan/SN10/2018 | 97% |
|  | M | A/swine/Henan/SN10/2018 | 99.7% |
|  | NS | A/swine/Henan/SN10/2018 | 99.9% |

**Fig. S1** Description of the clinical case. **(A)** The clinical symptom of miscarriage in pregnant sows. **(B)** Epidemiology of miscarriage in pregnant sows.


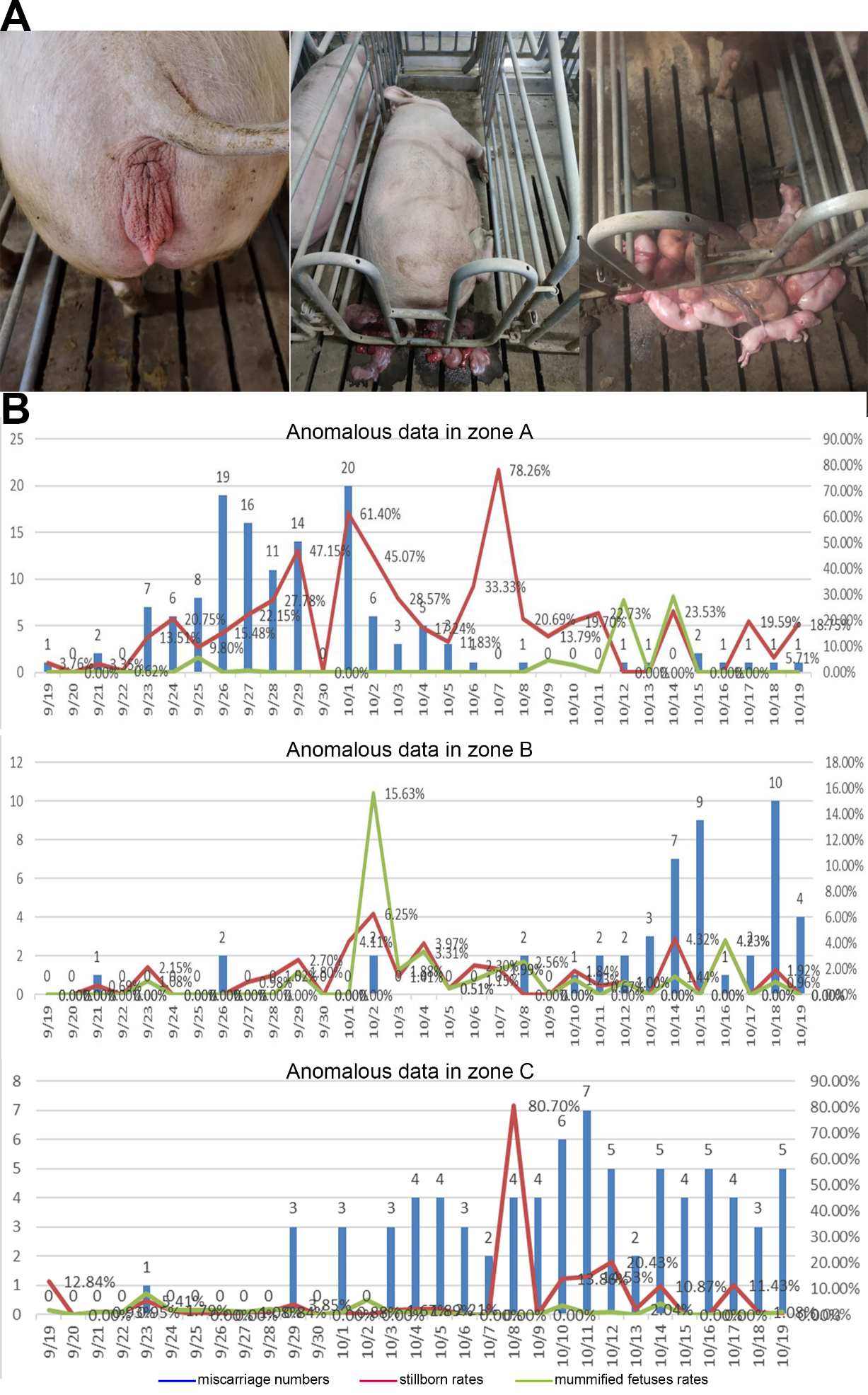


**Fig. S2** Identification of hemagglutination activity of the isolate virus. The virus P3-P5 generations were diluted at a 2-fold ratio and 30uL of virus was then added to 30uL of 1% chicken red blood cells to determine hemagglutination.


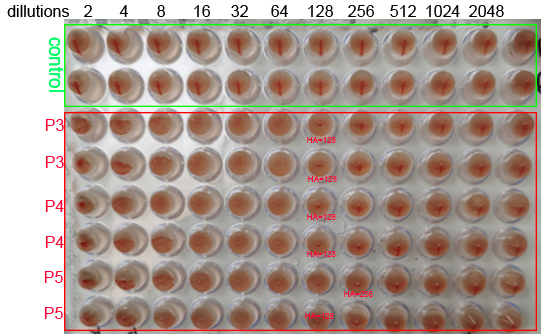


**Fig. S3** Phylogenetic analysis of the PB2 (A), PB1 (B), PA (C), NP (D), NA(E), M (F), and NS (G)genes. The trees were constructed by using the neighbor-joining method with the Maximum Composite Likelihood model and MEGA version 7.0 with 1,000 bootstrap replicates. The virus isolated in this study was indicated by purple triangle marker “▲”.

**Figure S3A**


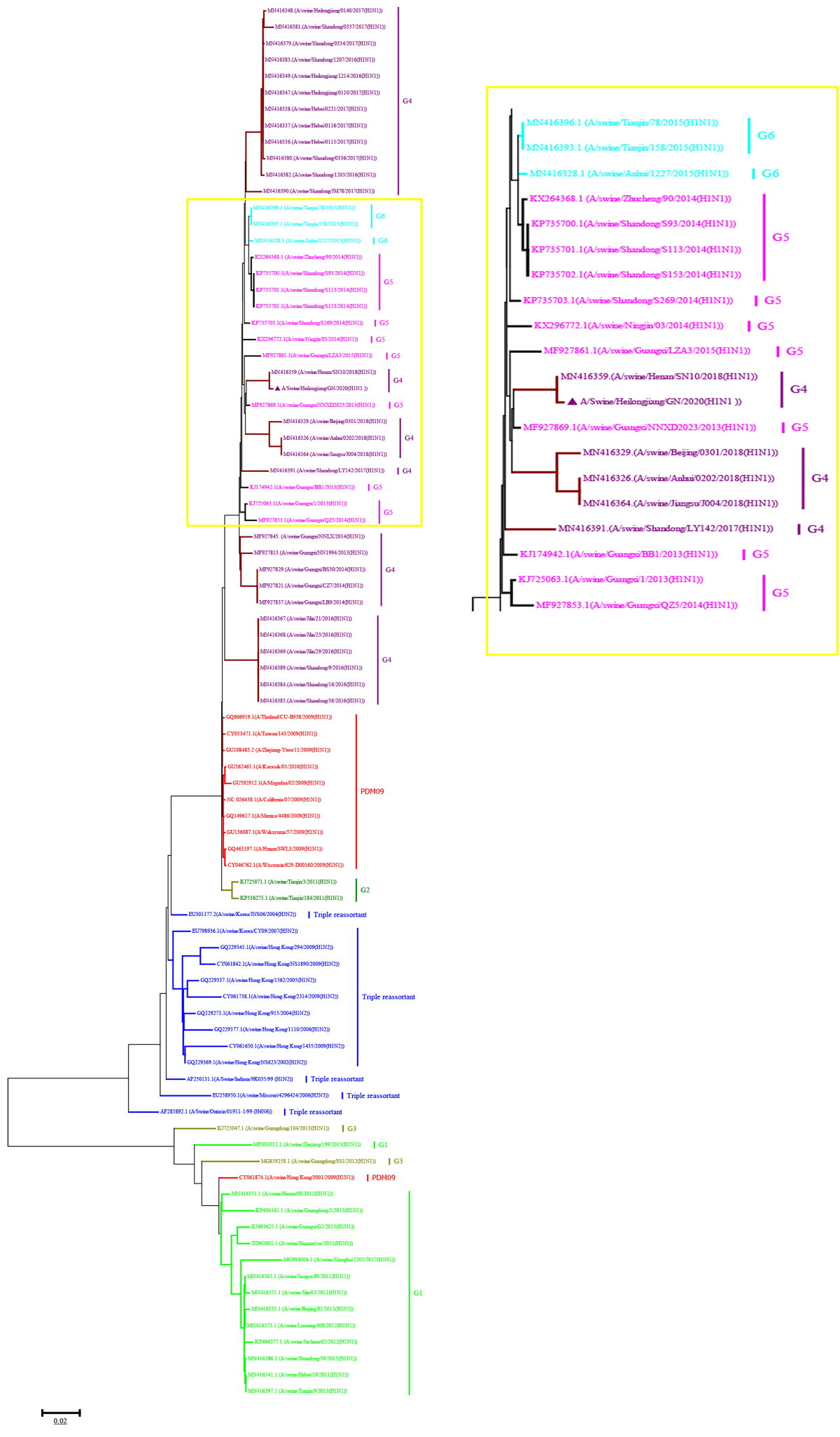


**Figure S3B**


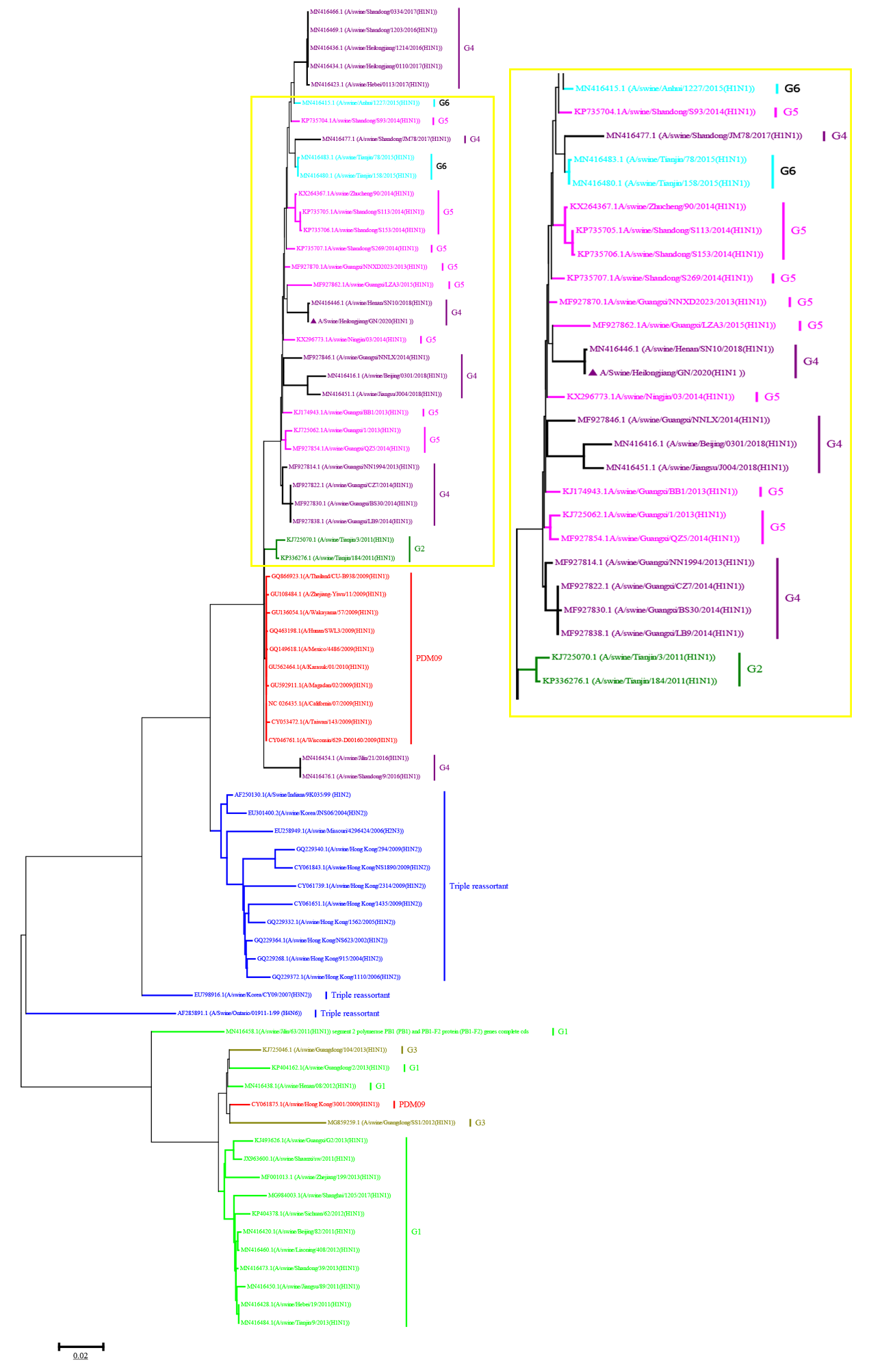


**Figure S3C**


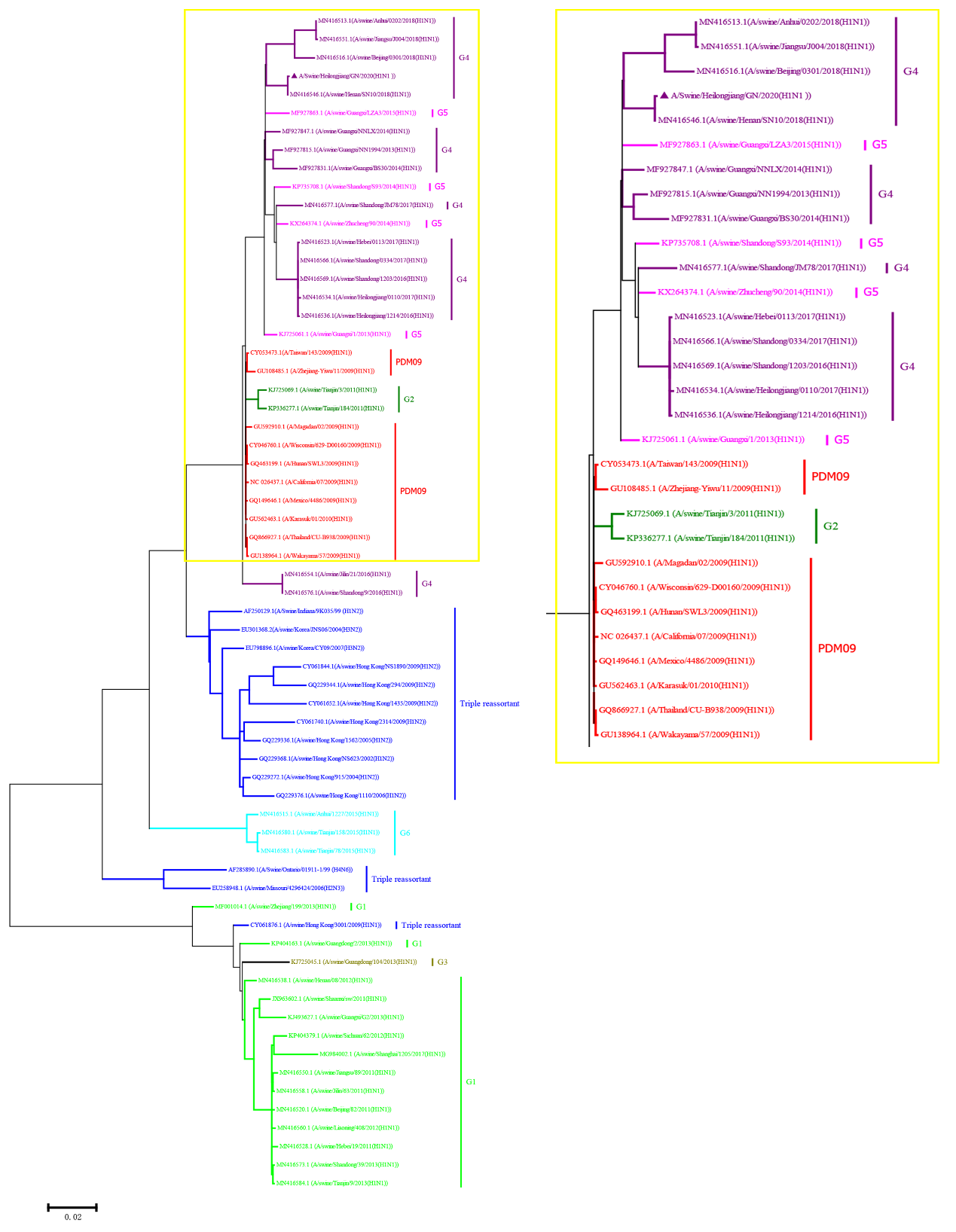


**Figure S3D**


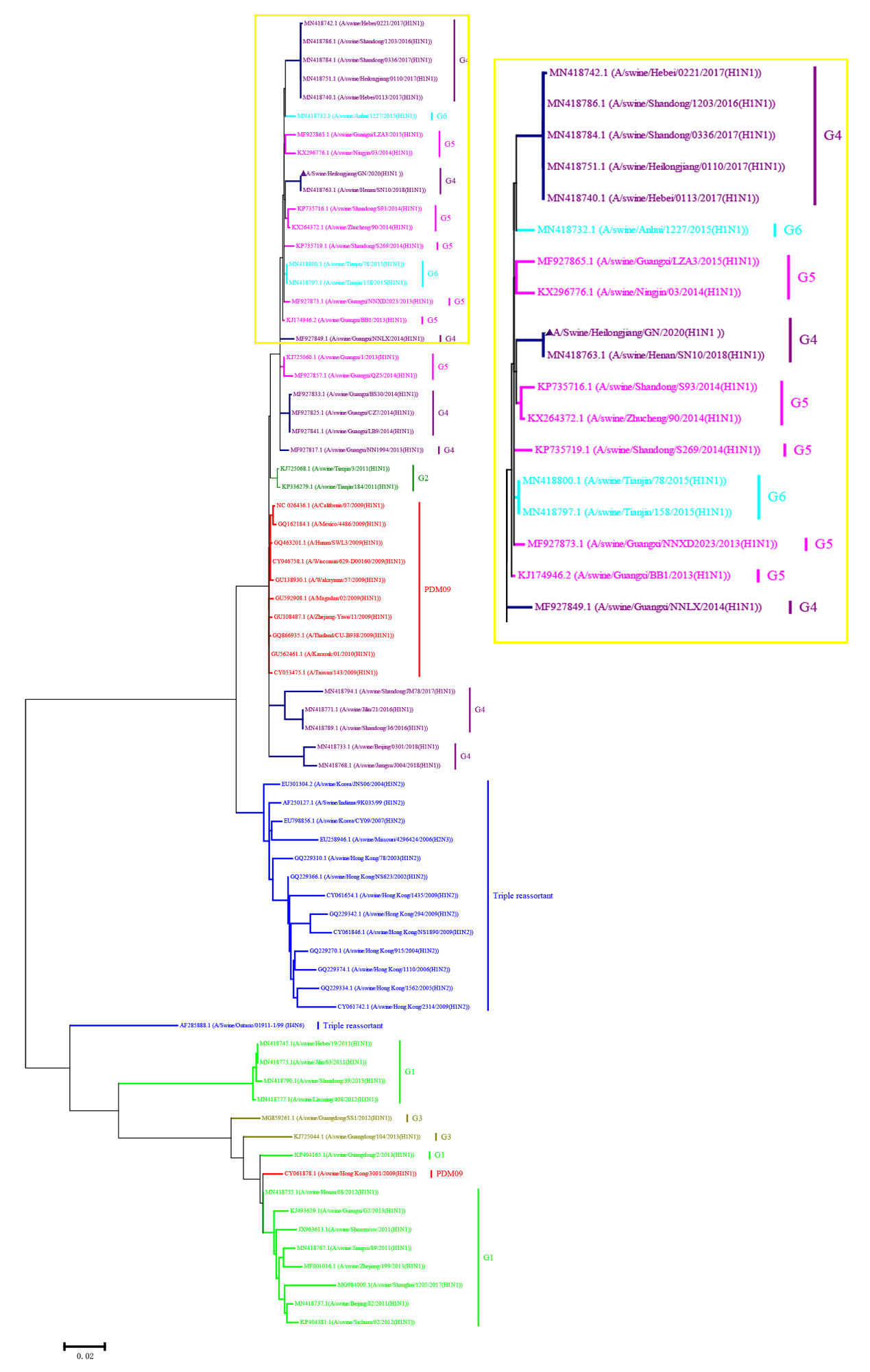


**Figure S3E**


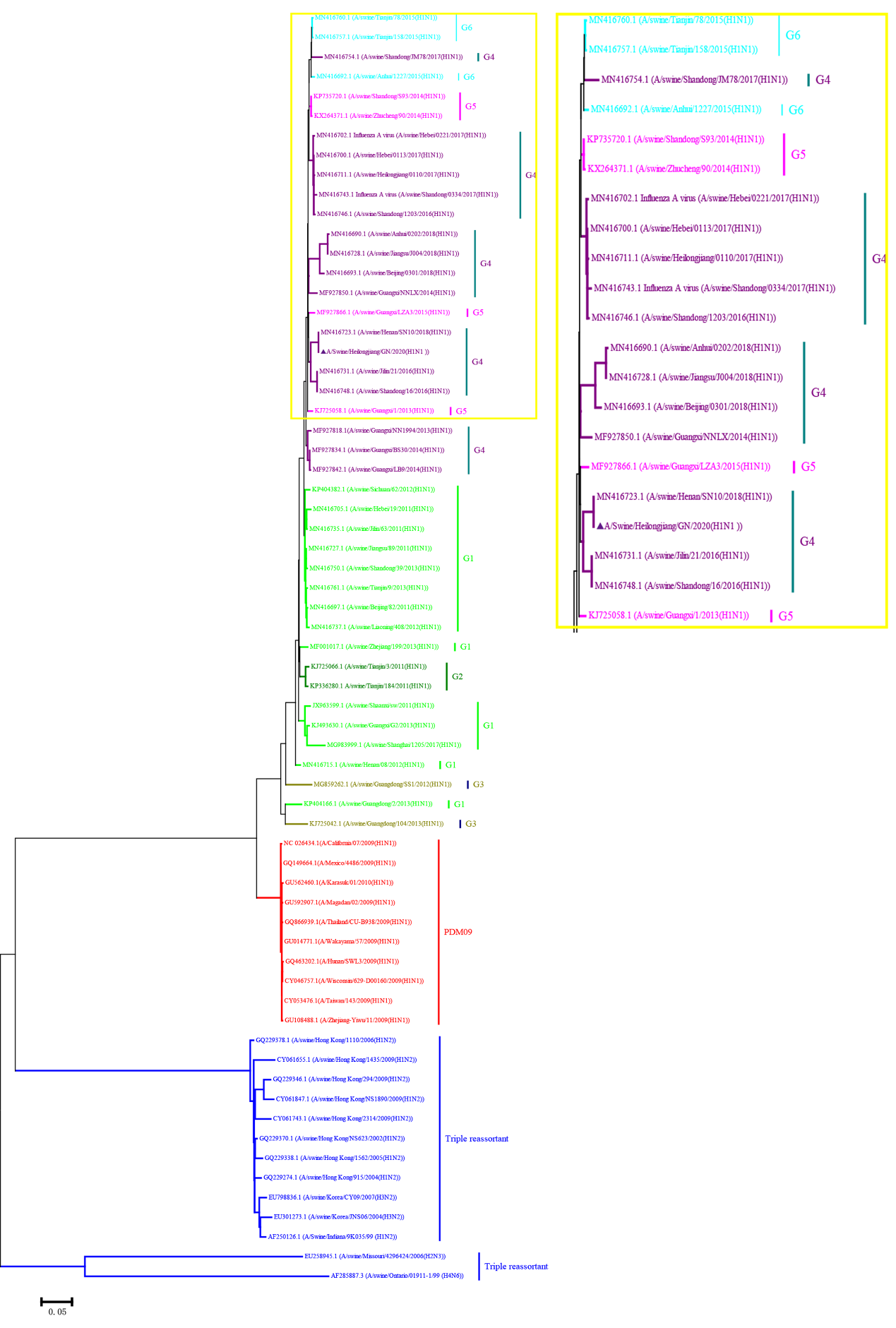


**Figure S3F**


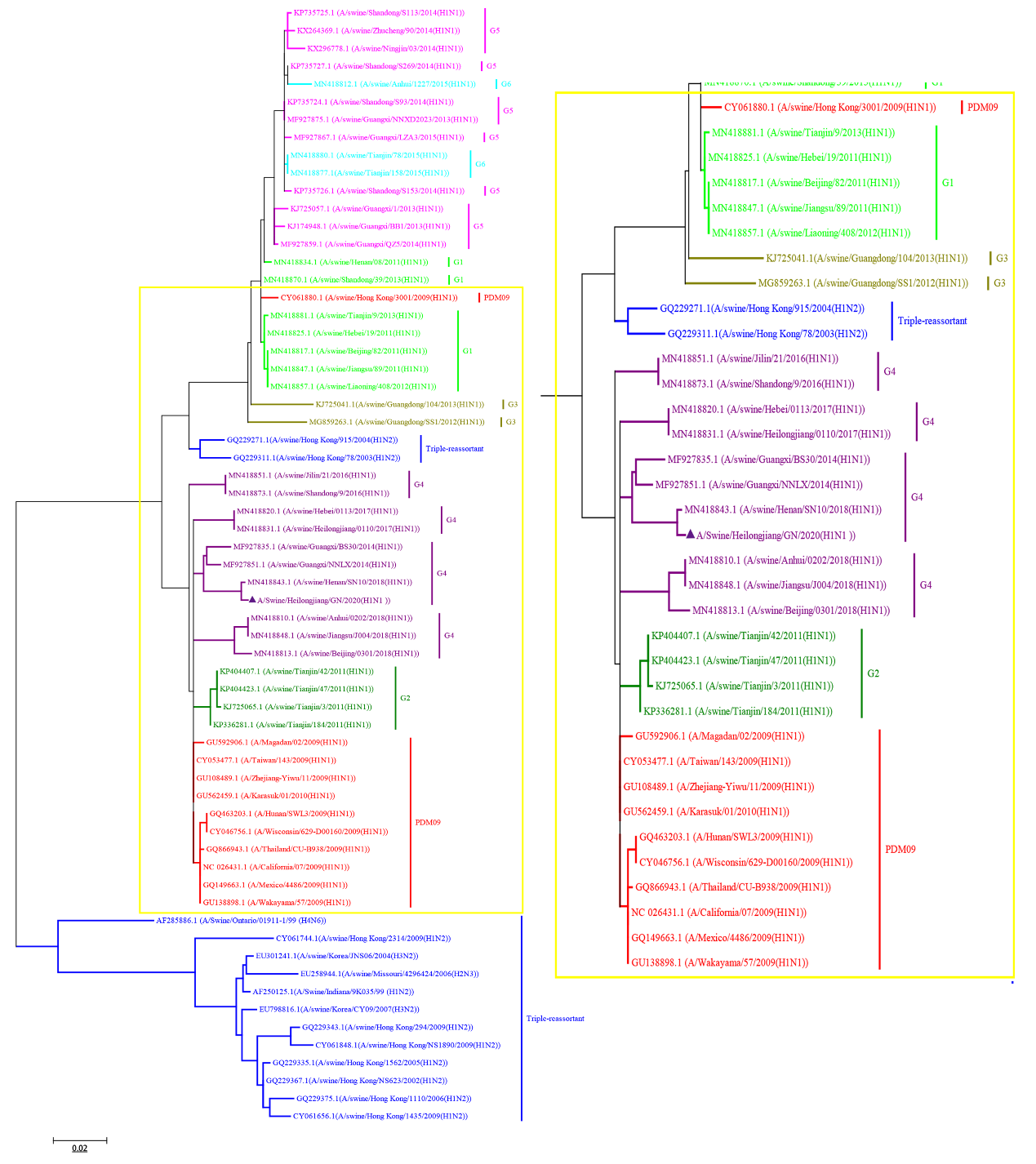


**Figure S3G**


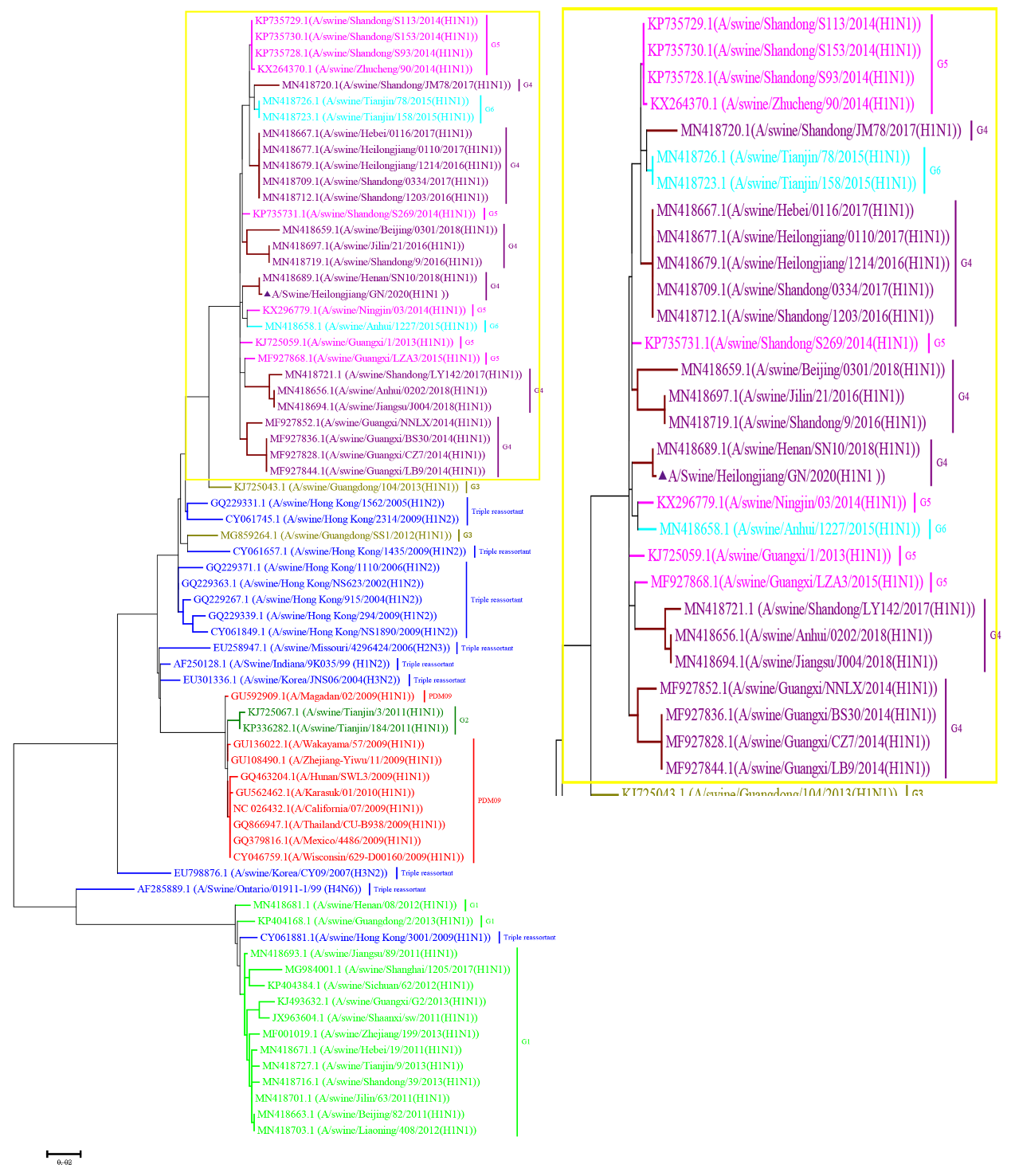


**Figure S4** The 50% mice lethal dose (MLD_50_) of A/swine/Heilongjiang/2020. The six-week-old female BALB/c mice were infected intranasally with 50 uL 101 to106 50% egg infectious dose virus (EID50) to detect MLD50.


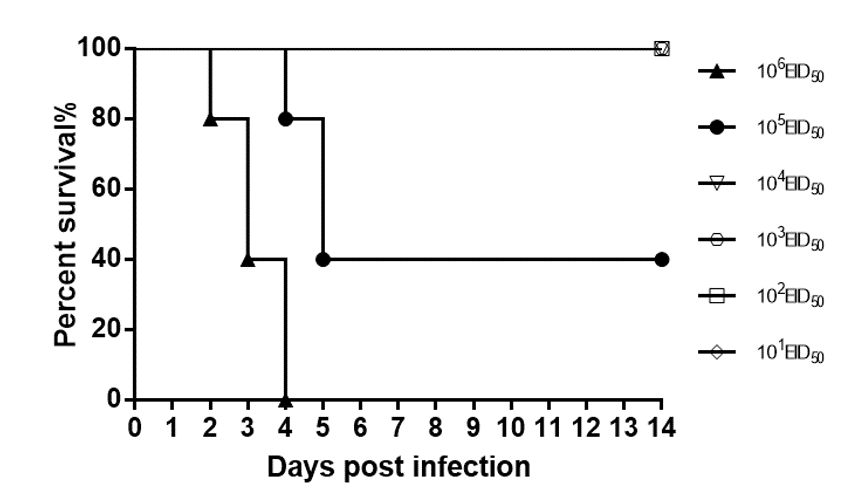

Supplement: Supplementary file 1 — Additional file 1: Table S1 The primers were used in our study. Table S2 The nucleotide homology between the A/swine/Heilongjiang/GN/2020 and A/swine/Henan/SN10/2018 virus, when analyzing each gene fragment. Fig. S1 Description of the clinical case. (A) The clinical symptom of miscarriage in pregnant sows. (B) Epidemiology of miscarriage in pregnant sows. Fig. S2 Identification of hemagglutination activity of the isolate virus. The virus P3-P5 generations were diluted at a 2-fold ratio and 30uL of virus was then added to 30uL of 1% chicken red blood cells to determine hemagglutination. Fig. S3 Phylogenetic analysis of the PB2 (A), PB1 (B), PA (C), NP (D), NA(E), M (F), and NS (G)genes. The trees were constructed by using the neighbor-joining method with the Maximum Composite Likelihood model and MEGA version 7.0 with 1,000 bootstrap replicates. The virus isolated in this study was indicated by purple triangle marker “▲”. Figure S4 The 50% mice lethal dose (MLD50) of A/swine/Heilongjiang/2020. The six-week-old female BALB/c mice were infected intranasally with 50 uL 101 to106 50% egg infectious dose virus (EID50) to detect MLD50. [file 12985_2022_1936_MOESM1_ESM.docx]
